# The Na^+^/H^+^ Exchanger-3 (NHE3) Activity Requires Ezrin Binding to Phosphoinositide and Its Phosphorylation

**DOI:** 10.1371/journal.pone.0129306

**Published:** 2015-06-04

**Authors:** Victor Babich, Francesca Di Sole

**Affiliations:** 1 Department of Medicine, University of Maryland School of Medicine, Baltimore, Maryland, United States of America; 2 Department of Internal Medicine, University of Texas Southwestern Medical Center, Dallas, Texas, United States of America; 3 Physiology and Pharmacology Department, Des Moines University, Iowa, United States of America; University of Geneva, SWITZERLAND

## Abstract

Na^+^/H^+^ exchanger-3 (NHE3) plays an essential role in maintaining sodium and fluid homeostasis in the intestine and kidney epithelium. Thus, NHE3 is highly regulated and its function depends on binding to multiple regulatory proteins. Ezrin complexed with NHE3 affects its activity via not well-defined mechanisms. This study investigates mechanisms by which ezrin regulates NHE3 activity in epithelial Opossum Kidney cells. Ezrin is activated sequentially by phosphatidylinositol-4,5-bisphosphate (PIP2) binding and phosphorylation of threonine 567. Expression of ezrin lacking PIP2 binding sites inhibited NHE3 activity (-40%) indicating that ezrin binding to PIP2 is required for preserving NHE3 activity. Expression of a phosphomimetic ezrin mutated at the PIP2 binding region was sufficient not only to reverse NHE3 activity to control levels but also to increase its activity (+80%) similar to that of the expression of ezrin carrying the phosphomimetic mutation alone. Calcineurin Homologous Protein-1 (CHP1) is part, with ezrin, of the NHE3 regulatory complex. CHP1-mediated activation of NHE3 activity was blocked by expression of an ezrin variant that could not be phosphorylated but not by an ezrin variant unable to bind PIP2. Thus, for NHE3 activity under baseline conditions not only ezrin phosphorylation, but also ezrin spatial-temporal targeting on the plasma membrane via PIP2 binding is required; however, phosphorylation of ezrin appears to overcome the control of NHE3 transport. CHP1 action on NHE3 activity is not contingent on ezrin binding to PIP2 but rather on ezrin phosphorylation. These findings are important in understanding the interrelation and dynamics of a CHP1-ezrin-NHE3 regulatory complex.

## Introduction

Ezrin is a member of the highly evolutionarily conserved ERM (Ezrin, Radixin and Moesin) protein family. It provides a regulated linkage between the plasma membrane and the actin cytoskeleton [1, 2]. Ezrin is composed of a globular domain located at its N-terminus followed by a predicted α-helical structure and approximately 100 amino acids of the C-terminal domain. The N-terminus is responsible for membrane targeting [2, 3] via binding to phosphatidylinositol 4,5-bisphosphate (PIP2), whereas the C-terminus binds actin [4]. Ezrin undergoes an intramolecular head-to-tail association in its inactive state and requires a direct interaction with PIP2 to open the closed conformation and the phosphorylation of threonine 567 (T567) to unmask the actin binding site [5]. Interaction with PIP2 occurs before ezrin phosphorylation to activate ezrin [6] and enhances ezrin binding to both plasma membrane-associated proteins and actin filaments [3, 7–12].

Upon reaching the plasma membrane, ezrin engages a number of membrane-associated proteins through its N-terminal domain [13, 14]. One such protein is the Na^+^/H^+^ exchanger-3 (NHE3) that is expressed on the brush border membrane of renal and intestinal epithelial cells [15, 16] and is of major importance in mediating Na^+^, Cl^-^, HCO_3_^-^ and fluid absorption in these epithelia [17–19]. NHE3 function requires an intact actin cytoskeleton [20, 21] and ezrin is proposed as the protein that provides this regulated linkage between NHE3 and the actin cytoskeleton. Indeed, NHE3 binds to ezrin both indirectly and directly through the cytoplasmic domain; indirect binding, via the PDZ-domain-containing protein the Na^+^/H^+^-exchanger regulatory factors (NHERF) 1 and 2 [22], mediates several aspects of NHE3 regulation [22, 23], while direct binding [24] affects many aspects of basal NHE3 trafficking [24, 25].

The Calcineurin Homologous Protein-1 (CHP1) is a widely expressed and highly conserved cytosolic Ca^2+^-binding protein [26]. It belongs to the EF-hand subfamily (calcium-binding motifs composed of E and F helixes joined by a loop) that is a very versatile class of proteins [27–29]. CHP1 interacts with several NHE isoforms including NHE3 [30–32] and regulates their function [26, 30, 31, 33]; in particular, CHP1 is a signal intermediate in the control of NHE3 activity under baseline and stimuli-mediated conditions [32, 34].

We previously demonstrated that CHP1, ezrin and NHE3 are linked in a regulatory complex [34]. Here, we analyzed which mechanisms of ezrin activation affect NHE3 activity. We found that ezrin binding to PIP2 and its phosphorylation at threonine 567 are both involved in controlling NHE3 activity under baseline conditions (Figure A in S1 File). Furthermore, we shed light on mechanisms of ezrin activation under CHP1 control. In previous studies, we determined that CHP1 expression affects ezrin phosphorylation [34], but changes in ezrin phosphorylation might be secondary to changes in ezrin binding to PIP2. A function of CHP1 in the regulation of ezrin binding to PIP2 is supported by the fact that Ca^2+^ binding proteins biochemically compete with phosphoinositide for the regulation of transport proteins [35, 36]. However, our observations did not support an action of CHP1 on ezrin by affecting its binding to PIP2 (Figure B in S1 File, pathway 1). Further studies are necessary to understand how CHP1 controls ezrin phosphorylation (Figure B in S1 File, pathway 2).

## Materials and Methods

### Reagents and primary antibodies

All chemicals were obtained from Sigma (St. Louis, MO, USA) except the cell culture reagents (Dulbecco modified Eagle medium, PBS, FCS, penicillin/streptomycin, and trypsin—EDTA) and the acetoxymethyl ester of 2′,7′-bis(carboxyethyl)-5(6)-carboxyfluorescein (BCECF-AM) that were obtained from Invitrogen (Carlsbad, CA, USA). The enhanced chemoluminescence system was from Amersham Biosciences (Piscataway, NJ, USA). The following primary antibodies were used, anti-ezrin rabbit polyclonal antibodies (Abcam, Cambridge, MA, USA) and β-actin mouse monoclonal antibody (Sigma, St. Louis, MO, USA).

### Cell Culture and Transient Transfection

Opossum Kidney (OK) cells, obtained from the American Type Culture Collection (ATCC, Manassas, VA, USA), were cultured in a mixture of Dulbecco modified Eagle medium (DMEM) supplemented with 10% FCS, 2 mM glutamine, 50 IU/ml penicillin and 50 μg/ml streptomycin. Cells were incubated in a humidified 95% air / 5% CO_2_ atmosphere at 37°C and sub-cultured weekly by trypsinization using 0.1% trypsin / 0.5 mM EGTA in PBS [32, 34, 37]. Cells generally reached confluence within 3 to 4 days, and confluent cells were serum-deprived for 2 days before assay. Studies on OK cells were performed between passages 23 and 57. OK cells were transiently transfected with plasmids using Lipofectamine 2000 (Invitrogen, Carlsbad, CA, USA) according to the manufacturer’s instructions. Transfection efficiency was monitored by transfection of the pEGFP-N1 vector (Clontech, Mountain View, CA, USA) and was approximately 60 to 70%.

### Plasmid Constructs

Full-length human ezrin was cloned into mEGFP-N1 (encoding a monomeric version of the enhanced green fluorescent protein (EGFP) [38]) and mutated at threonine 567 by site-directed mutagenesis [34]. Mutations in the PIP2 binding region of ezrin [11] were introduced by substituting four lysines (positions 253, 254, 262 and 263) with four asparagines via site-directed mutagenesis using the following primers K253N/K254N 5’-CAGGAACATCTCTTTCAATGACaataatTTTGTCATTAAACCCATCGACAAG-3’ and K262N/K263N 5’-GTTTGTCATTAAACCCATCGACaataatGCACCTGACTTTGTGTTTTATG-3’. Mutations were generated using the QuikChange II Site-Directed Mutagenesis kit (Agilent Technologies, Santa Clara, CA, USA) according to the manufacturer’s instructions. To produce the myc-tagged variants of ezrin and its mutants, the mEGFP-N1 vector (Clontech, Mountain View, CA, USA) was digested with AgeI and BsrGI and re-ligated with myc-containing oligonucleotide adaptor. Expression of the ezrin constructs did not cause detectable changes in cell shape [34]. His_6_ tagged full-length mouse CHP1-encoding cDNA was generated by digestion of CHP1-EGFP [34] with AgeI/BsrGI restriction enzymes. EGFP cDNA was extracted and substituted in frame with oligonucleotide adaptor corresponding to His_6_ tag [39]. All constructs were sequence-verified.

### Measurement of Na^+^/H^+^ Exchange Activity

NHE3 activity in OK cells was measured fluorometrically using the intracellularly trapped pH-sensitive dye BCECF-AM as previously described [34, 40]. Briefly, cells grown on glass coverslips were loaded with 10 μM BCECF-AM (30 minutes at 37°C) and pHi was estimated from the ratio of fluorescence in a computer-controlled spectrofluorometer (λ_excitation_: 500 and 450 nm, λ_emission_: 530 nm). The 500/450 nm fluorescence ratio was calibrated to pHi using K^+^/nigericin. Na^+^/H^+^ exchange activity was assayed as the initial rate of the Na^+^-dependent pHi increase after an acid load using nigericin in the absence of CO_2_/HCO_3_^−^. Comparisons were always made between cells of the same passage studied on the same day.

### Quantification of Membrane and Cytosol Antigen by Immunoblot

Quiescent confluent OK cells (48 hours in serum-free media) were washed with ice-cold PBS and gently scraped into membrane buffer (containing in mM: 150 NaCl, 50 Tris-HCl, pH 7.4, 5 EDTA, supplemented with protease inhibitors). Cells in suspension were homogenized by sonication and centrifuged at 10,000g for 10 minutes. The resultant supernatants were centrifuged at 100,000g for 30 minutes, cytosolic and crude membrane fractions were collected. The protein content of the crude membrane and cytosolic fractions were quantified by the Bradford method. An equal amount of proteins (50 μg) from both fractions were subjected to SDS-PAGE and blotted with either ezrin polyclonal antibodies (Abcam, Cambridge, MA, USA) or β-actin mouse monoclonal antibody (Sigma, St. Louis, MO, USA). Ezrin antigen signals were normalized to β-actin.

### Confocal Imaging

OK cells, plated on glass coverslips, were fixed (4% paraformaldehyde in PBS for 20 minutes), permeabilized (0.01% Triton X-100 in PBS for 5 minutes) and blocked in 1% BSA for 1 hour. Confocal fluorescence images were collected with a Zeiss 510 laser-scanning confocal microscope using a 100×/1.4NA objective lens. Fluorescence of mEGFP was excited by a 488 nm laser. Emitted light was separated by a beam splitter (TFT 395/495/610) and detected on a photomultiplier channel via an emission filter (Band Pass 535/30).

### Statistical Analysis

Results are represented as mean ± standard error. Quantitative differences between control and test conditions were assessed statistically by ANOVA. A probability (*P*) <0.05 was considered statistically significant. Four independent experiments per condition were analyzed if not otherwise stated.

## Results

### Cellular localization and sub-cellular fraction distribution of ezrin and its variants

**Fig 1:**
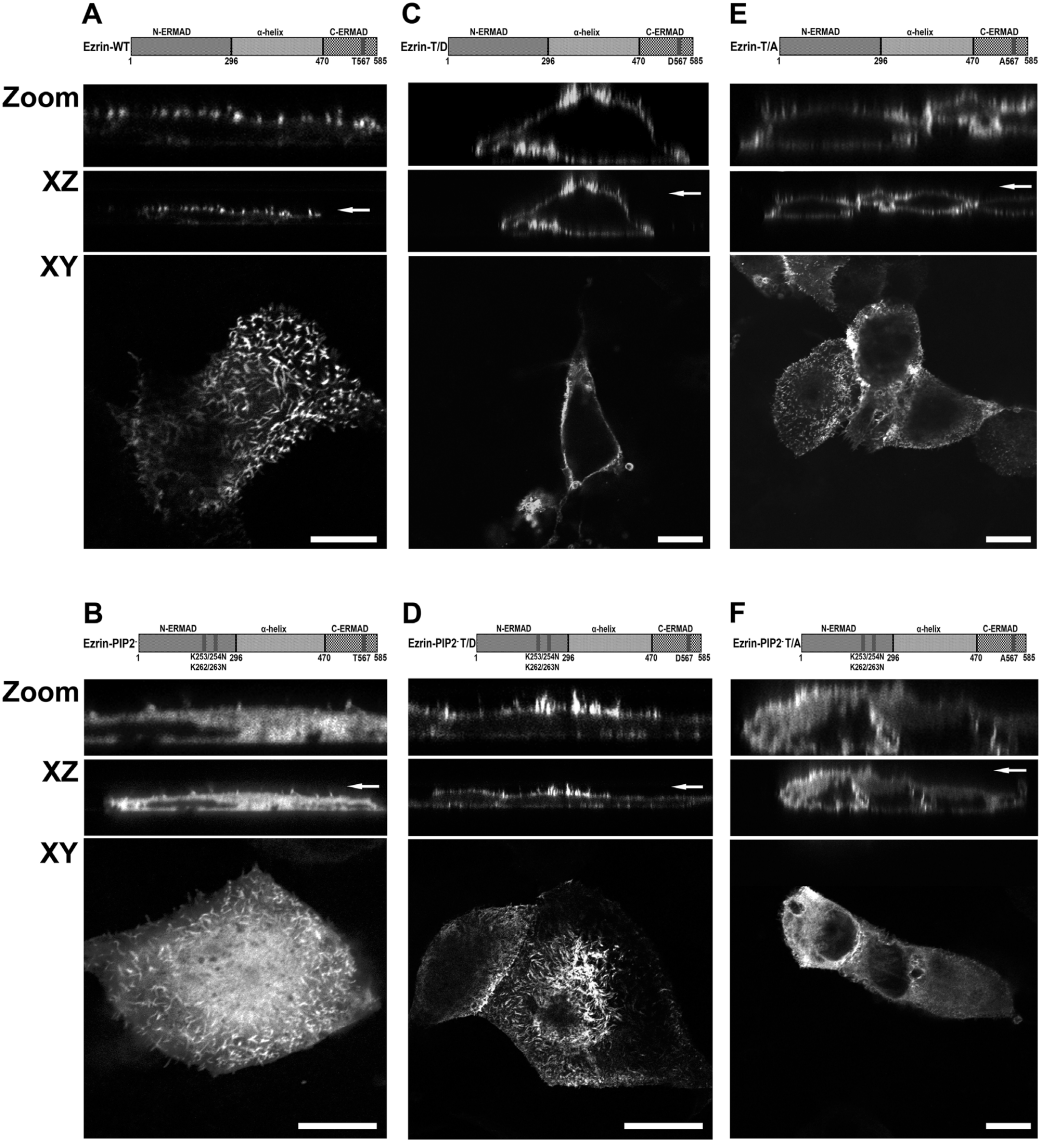
List of ezrin variants and their localization in OK cells. Six ezrin constructs were used: **A.** A full length wild-type ezrin (Ezrin-WT); **B.** An ezrin in which the binding to PIP2 was abolished by mutation of two double lysines to asparagines, K253/254N and K262/263N, (Ezrin-PIP2^-^ [6]); **C.** An ezrin with a threonine to aspartic acid point mutation at position 567 (Ezrin-T/D—phosphothreonine mimic, constitutive active [41]); **D.** An ezrin with T567D mutation in the PIP2^-^ ezrin mutant (Ezrin-PIP2^-^ T/D [6]); **E.** An ezrin with a threonine to alanine point mutation at position 567 (Ezrin-T/A—non phosphorylable that binds inefficiently to the actin cytoskeleton [41]); **F.** An ezrin with T567A mutation in the PIP2^-^ ezrin mutant (Ezrin-PIP2^-^ T/A [6]). The six ezrin constructs were mEGFP. Localization of ezrin and variants was visualized by indirect immunofluorescence (4 experiments per condition). Highlighted in the figure are XZ cross-sections (plus zoom) and XY face views. Apical localization is indicated by an arrow. Scale bar 2 μm.

Ezrin is activated by binding to PIP2 followed by phosphorylation of threonine 567 [6]. To investigate the relative contribution of these ezrin activating processes in the control of NHE3 activity, the function of six ezrin variants transiently expressed in Opossum Kidney (OK) cells were analyzed:

- A full length wild-type ezrin (Ezrin-WT, Fig 1A).
- An ezrin in which the binding to PIP2 is abolished by mutation of two double lysines to asparagines, K253/254N and K262/263N, (Ezrin-PIP2^-^, Fig 1B) [6].
- An ezrin with a threonine to aspartic acid point mutation at position 567 (T567D) (phosphothreonine mimic, constitutive active [41], Ezrin-T/D, Fig 1C).
- An ezrin with T567D mutation in the PIP2^-^ ezrin mutant (Ezrin-PIP2^-^ T/D, Fig 1D) [6].
- An ezrin with a threonine to alanine point mutation at position 567 (T567A) (non phosphorylatable that binds inefficiently to the actin cytoskeleton [41], Ezrin-T/A, Fig 1E).
- An ezrin with T567A mutation in the PIP2^-^ ezrin mutant (Ezrin-PIP2^-^ T/A, Fig 1F) [6].

**Fig 2:**
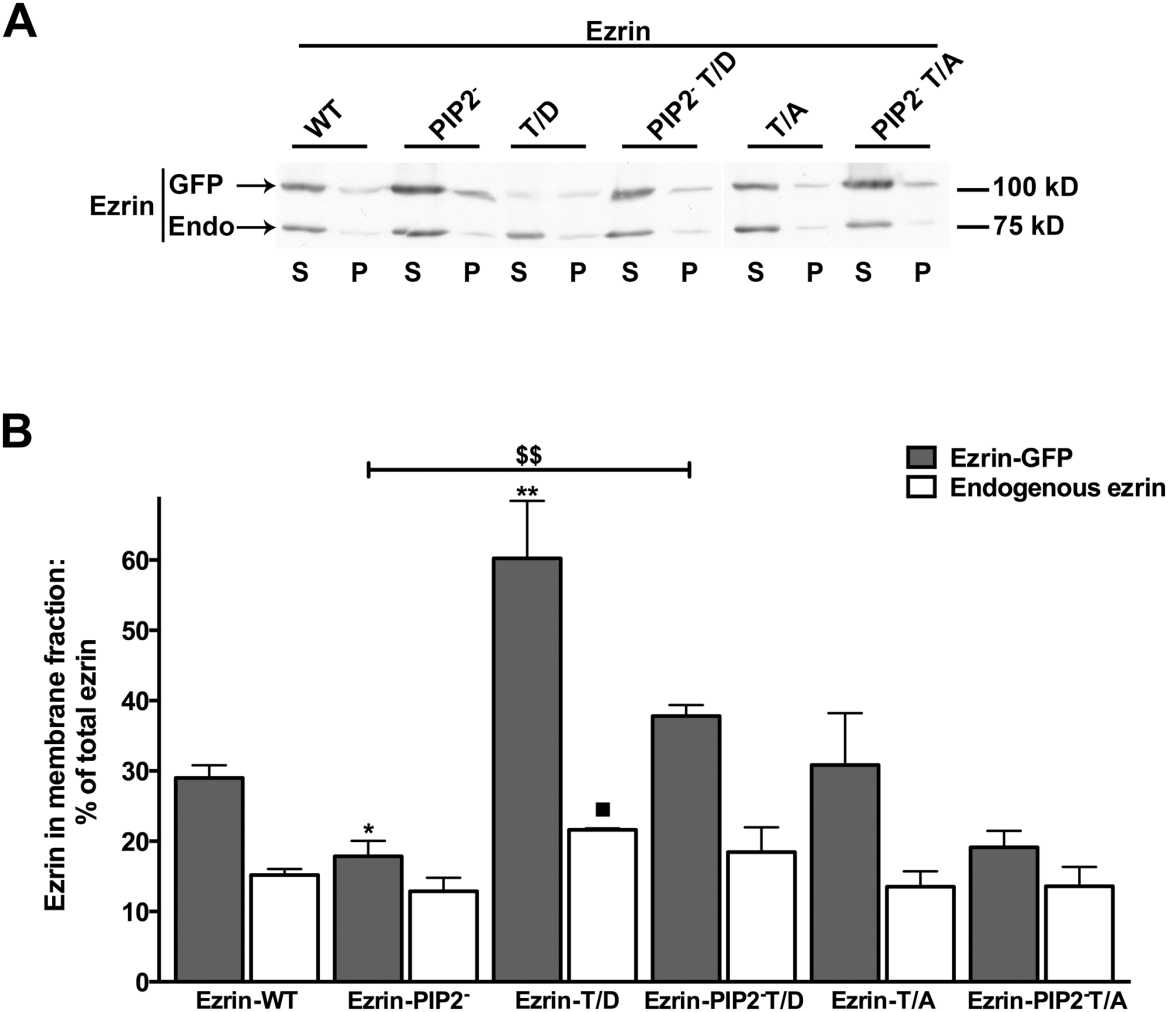
Distribution of ezrin and its variants in sub-cellular fraction. Cellular fractions were prepared from OK cells and equal amounts of proteins (50 μg/lane) from a membrane-enriched fraction (P, 100,000 x g pellet) and cytosol (S, 100,000 x g supernatant) were blotted and probed with anti-total ezrin. **A:** One of the immunoblots is shown. GFP = transfected mEGFP tagged ezrin (Ezrin-GFP) and Endo = endogenous ezrin. **B:** Statistical analysis of signal intensity ratios relative to the immuno-detected band in S and P is reported. The signal of total ezrin in S plus P fractions was defined as equal to 100% for each condition and results are expressed as percentage of total ezrin in the P vs. S fraction. Antigen signals were normalized to β-actin. Bars and error bars represent the means ± standard errors (SE), respectively (4 experiments per condition). *P<0.05/** P<0.01 ANOVA, the membrane fraction of tagged ezrin in the presented groups compared to the membrane fraction of tagged ezrin in cells transfected with Ezrin-WT. ^■^P<0.05 ANOVA, membrane fraction of endogenous ezrin in the presented groups compared to the membrane fraction of endogenous ezrin in cells transfected with Ezrin-WT. ^$$^P<0.01 ANOVA membrane fraction of tagged ezrin in Ezrin-PIP2^-^ compared to Ezrin-PIP2^-^ T/D transfected cells.

The six ezrin constructs were mEGFP or c-myc tagged and localization of mEGFP tagged constructs was visualized (Fig 1). Ezrin-WT was localized mainly on the plasma membrane, while Ezrin-PIP2^-^ seemed uniformly expressed in OK cells (Fig 1A and 1B). Ezrin-T/D had a pattern of expression similar to Ezrin-WT (Fig 1C). Both constructs were expressed mainly at the apical membrane of OK cells, though Ezrin-T/D was expressed also at the basolateral membrane as was previously shown [34]. Interestingly, Ezrin-PIP2^-^ cellular localization changed when the threonine 567 was mutated to aspartic acid, Ezrin-PIP2^-^ T/D (Fig 1D); Ezrin-PIP2^-^ T/D localization resembled that of Ezrin-T/D indicating that mimicking ezrin phosphorylation was sufficient to secure a predominant cell surface expression of ezrin. Ezrin-T/A was also recruited to the plasma membrane but did not exclusively localize on the cell surface (Fig 1E and [34]). Mutation of threonine 567 to alanine did not change Ezrin PIP2^-^ cellular localization, Ezrin-PIP2^-^ T/A (Fig 1F). Constructs tagged with c-myc had a similar localization to the mEGFP tagged constructs (not shown). To test further the cellular localization of the ezrin constructs a membrane-enriched (P) fraction was separated from a cytosol (S) fraction in OK cells and equal amounts of proteins from both fractions were probed for total ezrin (Fig 2A and 2B). This set of experiments revealed that, indeed, the Ezrin-T/D was quantitatively more present in the membrane-enriched fraction and Ezrin-PIP2^-^ (similarly to Ezrin-PIP2^-^ T/A) was quantitatively less present in the membrane-enriched fraction compared to Ezrin-WT. Furthermore, the expression level of endogenous ezrin and its sub-cellular distribution were not significantly affected by expression of the ezrin variants; the exception was the expression of Ezrin-T/D that induced an increase in endogenous ezrin in the membrane-enriched fraction.

### Effect of ezrin activation by binding to PIP2 and phosphorylation of threonine 567 on NHE3 activity

**Fig 3:**
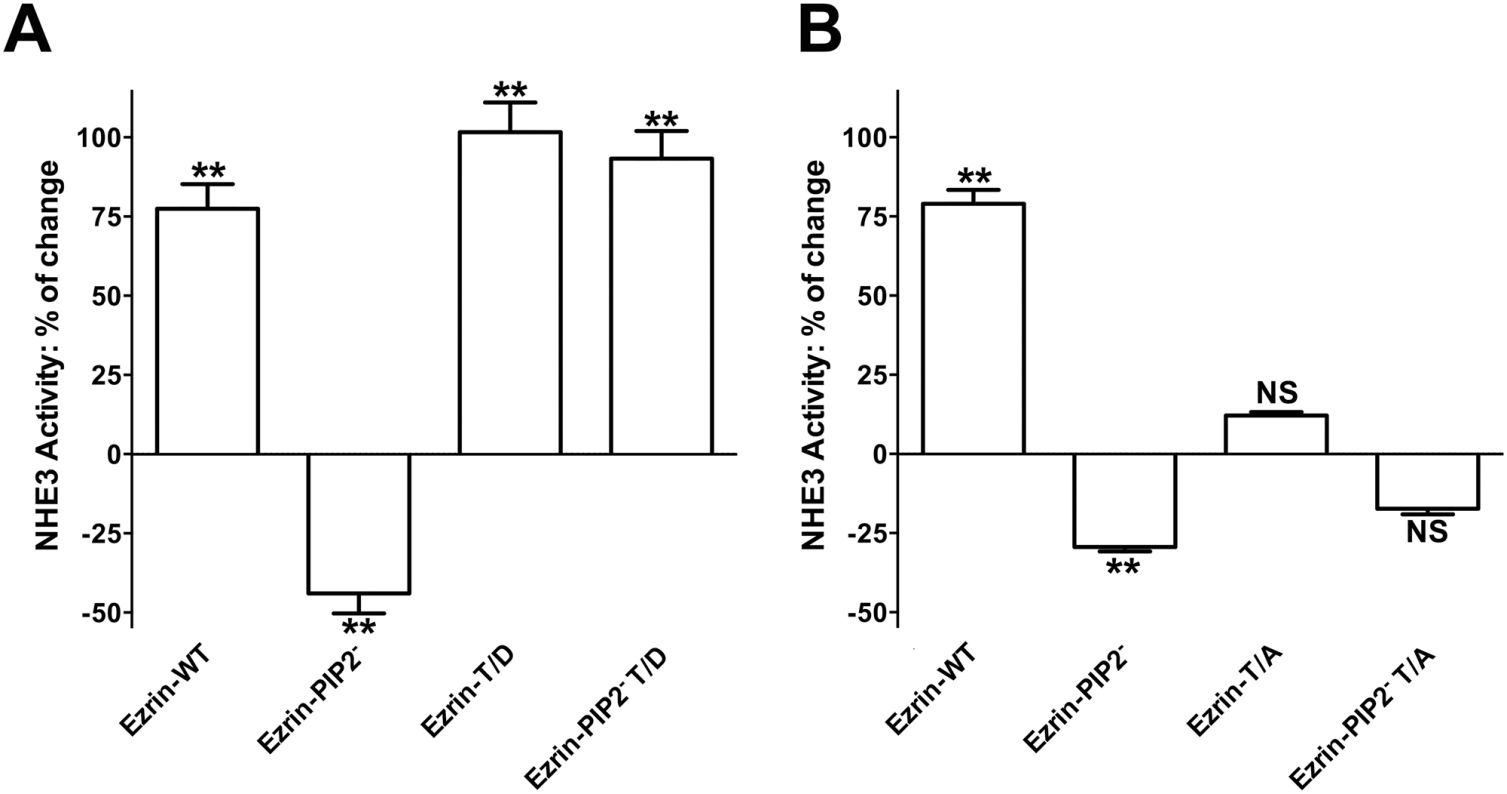
Effect of ezrin variants in PIP2 binding and 567 phosphorylation sites on NHE3 activity. NHE3 activity was measured as the rate of Na^+^-dependent intracellular pH recovery. Results are expressed as percentage of change in NHE3 activity. Bars and error bars represent the means and SE, respectively (4 experiments per condition). **P < 0.01 ANOVA compare to control (empty-vector-transfected cells). NS: Statistically insignificant compare to control. Effect of expression of **A.** Ezrin-WT, Ezrin-PIP2^-^, Ezrin-T/D and Ezrin-PIP2^-^ T/D. **B.** Ezrin-WT, Ezrin-PIP2^-^, Ezrin-T/A and Ezrin-PIP2^-^ T/A on NHE3 activity.

Expression of Ezrin-WT in OK cells increases NHE3 activity (+70%) (Fig 3A and 3B) as previously reported [34]. This effect on NHE3 activity was enhanced by expression of the constitutively active phosphothreonine mimic ezrin, Ezrin-T/D, (Fig 3A); whereas the inactive non-phosphorylatable ezrin mutant, Ezrin-T/A, did not affect NHE3 activity (Fig 3B). The increase in NHE3 activity induced by Ezrin-T/D was not significantly different from the increase induced by Ezrin-WT expression, possibly because the increase in NHE3 activity induced by expression of the constructs is close to an ezrin-dependent exchanger maximum activity. These findings support an earlier study [34]; specifically, NHE3 activity is not affected by ezrin expression *per se* [34, 42] but rather by its activated state. Ezrin-T/D is constitutive active, while Ezrin-WT is activated by phosphorylation. Indeed, Ezrin-WT was recognized by an anti-phospho-ezrin antibody when expressed in OK cells [34]. Furthermore, Ezrin-T/D has been associated with the formation of longer and more abundant microvilli [41]; hence, with an increase in cell surface area. Although, we cannot exclude an increase in cell surface area in the conditions under study, the fact that both Ezrin-WT and Ezrin-T/D expression activated NHE3 activity suggests that their effect on NHE3 is not due to a change in cell surface area.

**Fig 4:**
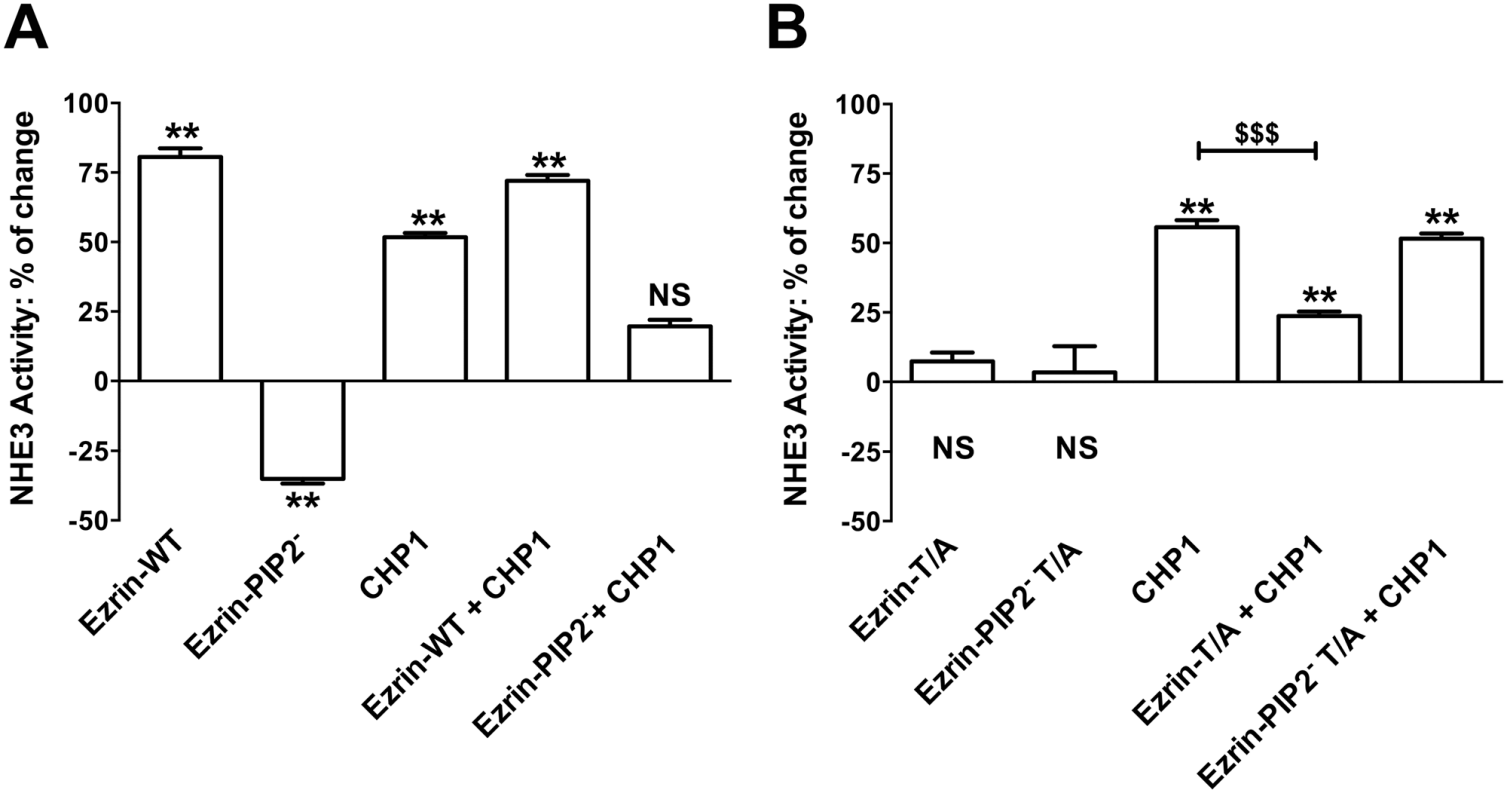
Effect of CHP1 expression on the ezrin-mediated control of NHE3 activity. NHE3 activity was measured as described in the legend of Fig 3. Bars and error bars represent the means and SE, respectively (8 experiments per condition). **P < 0.01 ANOVA compare to control (empty-vector-transfected cells), NS: Statistically insignificant compare to control. ^$$$^P<0.001 ANOVA, CHP1 expression compared to CHP1 and Ezrin-T/A co-expression. Effect of expression of **A.** Ezrin-WT, Ezrin-PIP2^-^, CHP1, CHP1 + Ezrin-WT or CHP1 + Ezrin-PIP2^-^. **B.** Ezrin-T/A, Ezrin-PIP2^-^ T/A, CHP1, CHP1 + Ezrin-T/A or CHP1 + Ezrin-PIP2^-^ T/A on NHE3 activity.

Because ezrin binding to PIP2 is necessary for its subsequent phosphorylation, we determined next whether expression of ezrin lacking the PIP2 binding site (Ezrin-PIP2^-^) affected NHE3 activity. Expression of Ezrin-PIP2^-^ significantly inhibited NHE3 activity (-40%) (Fig 3A) so indicating that the binding of ezrin to PIP2 is important for maintaining NHE3 activity under baseline conditions. Interestingly, the introduction of the T567D mutation in the Ezrin-PIP2^-^ mutant (Ezrin-PIP2^-^ T/D) was sufficient not only to reverse NHE3 activity to the control level, but also to increase it (+80%) (Fig 3A). Whereas Ezrin-PIP2^-^ seems to localize primarily in the cytoplasm, Ezrin-PIP2^-^ T/D was expressed on the plasma membrane of OK cells similarly to ezrin carrying the threonine 567 to aspartic acid mutation alone (Figs 1B, 1C, 1D and 2B). It suggested that the T567D mutation, and by implication threonine 567 phosphorylation, restores the capacity of Ezrin-PIP2^-^ to locate mainly on the plasma membrane and activate NHE3 activity under baseline conditions. Along these lines, the introduction of the T567A mutation in the Ezrin-PIP2^-^ mutant (Ezrin-PIP2^-^ T/A) was sufficient to render insignificant the effect on NHE3 activity induced by Ezrin-PIP2^-^ expression (Figs 3 and 4).

### Effect of CHP1 expression on ezrin activation to control NHE3 activity

**Fig 5:**
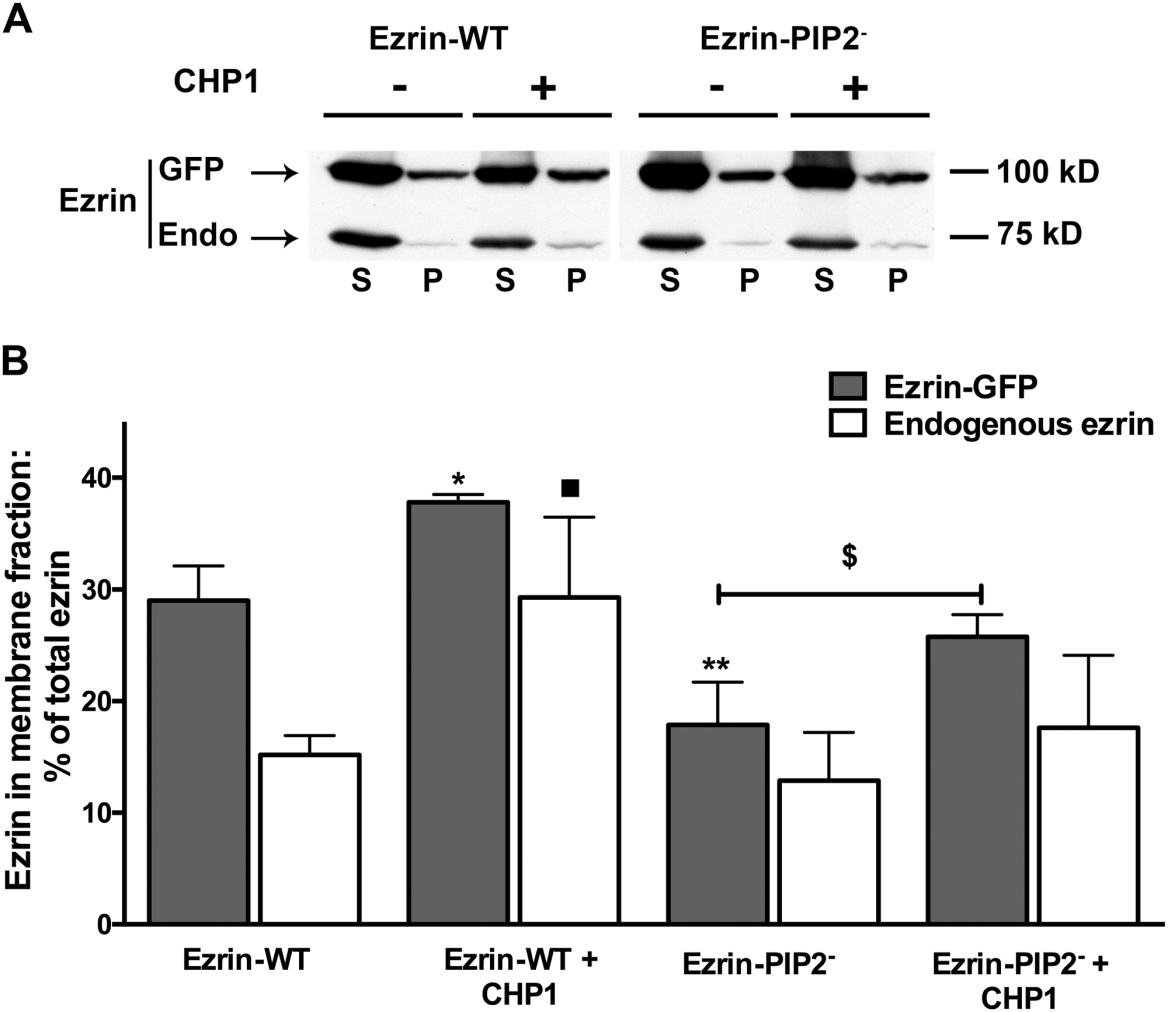
Effect of CHP1 expression on Ezrin-WT or Ezrin-PIP2^-^distribution in sub-cellular fraction. Cellular fractions were prepared as described in the legend of Fig 2. **A:** One of the immunoblots is shown. GFP = transfected mEGFP tagged ezrin (Ezrin-GFP) and Endo = endogenous ezrin. **B:** Statistical analysis of signal intensity ratios relative to the immuno-detected band in S and P is reported. The signal of total ezrin in S plus P fractions was defined as equal to 100% for each condition and results are expressed as percentage of total ezrin in the P vs. S fraction. Antigen signals were normalized to β-actin. Bars and error bars represent the means ± SE, respectively (4 experiments per condition). *P<0.05/**P < 0.01 ANOVA, membrane fraction of tagged ezrin in the presented groups compared to the membrane fraction of tagged ezrin in cells transfected with Ezrin-WT. ^■^P<0.05 ANOVA, membrane fraction of endogenous ezrin in the presented groups compared to the membrane fraction of endogenous ezrin in cells transfected with Ezrin-WT. ^$^P<0.05 ANOVA, membrane fraction of tagged ezrin in Ezrin-PIP2^-^ compared to Ezrin-PIP2^-^ + CHP1 transfected cells.

We have recently shown that CHP1 expression increased NHE3 transport activity (+50%) and ezrin phosphorylation at threonine 567 without changes in total ezrin whereas CHP1 silencing decreased NHE3 activity (-50%) and ezrin phosphorylation [34]. Furthermore, CHP1 knock-down reverses NHE3 activation induced by Ezrin-WT expression but not that induced by Ezrin-T/D [34]. Because linkage of ezrin to the plasma membrane occurs before its phosphorylation and actin cytoskeleton interaction [6], the effect of an ezrin construct lacking PIP2 binding on CHP1-dependent activation of NHE3 activity was determined (Fig 4A and 4B). The increase in NHE3 transport induced by co-expression of CHP1 and Ezrin-WT was similar to the one induce by expression of Ezrin-WT alone (+70%) (Fig 4A), confirming that CHP1 and ezrin stimulated NHE3 activity via a common signal cascade. However, when CHP1 was co-expressed with Ezrin-PIP2^-^ (which *per se* inhibits NHE3 by -40%) NHE3 activity was increased only by +10% (Fig 4A). It is of note that comparing the percentage of change in NHE3 activity induced by expression of Ezrin-PIP2^-^ (-40%) with that induced by expression of CHP1 and Ezrin-PIP2^-^ (+10%) in OK cells demonstrated an overall increase in transport activity of 50%, which is comparable to activation of NHE3 by CHP1 alone. This indicates that ezrin binding to PIP2 may not be central in the CHP1-dependent activation of NHE3. In support of this postulate, co-expression of CHP1 and Ezrin-T/A reduced NHE3 activity when compared to the expression of CHP1 alone (Fig 4B) whereas, co-expression of CHP1 and Ezrin-PIP2^-^ T/A increased NHE3 activity to a similar extent as the expression of CHP1 alone (Fig 4B). Ezrin-T/A and Ezrin-PIP2^-^ T/A *per se* had no effect on NHE3 activity (Figs 3B and 4B). These results indicate that Ezrin-T/A does compete with endogenous ezrin to affect the CHP1 induced activation of NHE3 while Ezrin-PIP2^-^ T/A does not. The cellular fractionation experiments supported these results. Indeed, CHP1 expression induced an increase in endogenous or exogenous ezrin in the membrane-enriched fraction (Fig 5A and 5B). Furthermore, CHP1 expression partially recovers the decrease in exogenous ezrin amount in the membrane-enriched fraction of OK cells expressing Ezrin-PIP2^-^ (Fig 5A and 5B).

In summary, CHP1 action to activate ezrin seems to be upstream of (or dependent on) ezrin phosphorylation and downstream of (or independent from) ezrin binding to PIP2 because CHP1 expression acts to compensate ezrin lacking binding to PIP2 but not ezrin lacking phosphorylation at threonine 567, i.e. the role of CHP1 in the control of NHE3 activity seems connected to anchoring ezrin to actin rather than ezrin to the plasma membrane.

## Discussion

Ezrin is a microfilament-membrane linker that provides linkage between the plasma membrane-associated protein, NHE3, and the actin cytoskeleton [1, 14] to regulate several aspects of NHE3 functions [24, 25, 34, 43, 44]. Specifically, direct binding of ezrin to NHE3 contributes to NHE3 activity by affecting newly synthesized NHE3 delivery to the plasma membrane and NHE3 brush border mobility [24].

In this study, to better understand the cellular mechanisms by which ezrin influences NHE3 function, both the PIP2 binding to and phosphorylation of ezrin and their possible synergism in the maintenance of NHE3 activity under baseline conditions were analyzed. Ezrin binding to PIP2 and its phosphorylation at threonine 567 rather than ezrin expression level *per se* [34, 42] were both involved in the control of NHE3 activity (Figure A in S1 File). CHP1 is together with ezrin part of the NHE3 regulatory complex [34]. Interestingly, the CHP1 role in controlling NHE3 basal activity [34, 39] appears to be dependent upon ezrin phosphorylation rather than on ezrin binding to PIP2 (Figure B in S1 File). This conjecture is based on the following findings.

Firstly, lack of ezrin binding to PIP2 inhibited NHE3 activity, suggesting that the ezrin mutant lacking PIP2 binding competes with endogenous ezrin molecules to maintain NHE3 basal activity. PIP2 is particularly located in subcellular structures on the apical membrane of epithelial cells such as the microvilli [45]; ezrin binding to PIP2 may serve for spatial-temporal targeting of the assembly and disassembly of protein complexes (e.g., kinases and co-factors) required for optimal NHE3 basal activity. Indeed, interaction of ezrin with PIP2 is believed to enhance ezrin binding to both plasma membrane-associated proteins and actin filaments [3, 7–12]. Of note, transport protein regulation by phosphoinositide is not surprising; NHE activity requires phosphoinositide association. Two functionally distinct phosphoinositide binding regions were identified on the exchanger C-terminus and their mutation led to the inhibition of the basal activity of both NHE1 and NHE3 [46, 47]. An inhibition of NHE3 transport capability induced by the expression of ezrin lacking the PIP2 binding domain might be an unrecognized phosphoinositide-dependent mechanism that requires ezrin as an intermediate.

Secondly, the T567D mutation not only activated NHE3 activity *per se*, but also restored the membrane cytoskeleton linker capacity of the ezrin mutated at PIP2 binding site to activate NHE3 activity. Indeed, while the Ezrin-PIP2^-^ mutant was found to be restricted to the cytoplasm (Fig 1B), the Ezrin-PIP2^-^ T/D mutant was recruited to the plasma membrane, similarly to ezrin carrying the T567D mutation alone (Fig 1C and 1D). These observations indicate that the phosphomimetic mutant is capable of controlling NHE3 basal activity counteracting the lack of ezrin binding to PIP2, which is in line with previous findings [6].

Thirdly, the T567A mutation, either alone or in combination with the mutation of the ezrin PIP2 binding site, did not affect NHE3 activity so confirming that the expression of ezrin *per se* is not sufficient to control NHE3 activity [34, 42]. Furthermore, contrary to Ezrin-PIP2^-^ expression, Ezrin-PIP2^-^ T/A did not inhibit NHE3. These findings support the postulate that although ezrin targeting to the plasma membrane by PIP2 binding is necessary for NHE3 basal activity, ezrin phosphorylation at threonine 567 is able to offset the effect of the lack of ezrin PIP2-binding in the control of NHE3 transport.

Fourthly, CHP1 plays a role in the ezrin-mediated control of NHE3 activity because CHP1 expression stimulates localization of endogenous and exogenous ezrin in a membrane-enriched fraction where most of the Ezrin-T/D was found and CHP1-dependent activation of the NHE3 activity was significantly reduced by the ezrin T567A mutation. These data are in agreement with our previous observations of CHP1 signaling upstream to ezrin phosphorylation in the modulation of NHE3 function [34]. CHP1 is probably an example of Ca^2+^-modulated regulatory proteins that intervenes in the fine-tuning of a relative large number of specific intracellular activities with functional protein interactions being the determinants for the activities of this class of regulatory proteins [26, 48]. Members of the EF hand Ca^2+^ binding protein family have been identified as ezrin ligands that specifically interact with the N-terminal domain of inactive ezrin to unmask the actin binding site [49, 50]. Although this study has not specifically addressed the interaction of CHP1 to ezrin as the mechanism by which CHP1 affects ezrin activation, our findings match those showing functional targeting of ezrin by Ca^2+^-modulated regulatory proteins. Furthermore, CHP1 is known to modulate either protein kinase or phosphatase activity [26, 51, 52] while several kinases and phosphatases are found to regulate ERM protein phosphorylation [53–59]. Concerning a possible role of CHP1 in the control of ezrin association with PIP2, EF hands Ca^2+^ binding proteins have been found to biochemically compete with phosphoinositide for the regulation of transport proteins [35, 36]. Our findings do not support this pathway (Figure B is S1 File, pathway 1). CHP1-mediated activation of NHE3 activity was not affected by mutation of ezrin at the PIP2 binding site. CHP1 expression induced a significant increase in Ezrin-PIP2^-^ localization in a membrane-enriched fraction, comparable to that induced in cells expressing Ezrin-WT, so confirming a CHP1 action independent of ezrin binding to PIP2. However, our results do not exclude an action of CHP1 on the regulation of the NHE3 transport by the direct association of phosphoinositide.

In summary, expression of ezrin *per se* is not sufficient to control NHE3 activity. However, a conformational change (activation) of ezrin in response to PIP2 binding and phosphorylation of threonine 567 is necessary to affect NHE3 basal activity. CHP1 is a signal intermediate proposed to act as a regulatory partner of ezrin possibly to support the creation of a microfilament environment for NHE3 function. Further studies are necessary to determine how CHP1 controls ezrin phosphorylation and ezrin binding to actin (Figure B in S1 File, pathway 2) and whether CHP1 and ezrin coordinate function targets specifically NHE3 or also other NHE isoforms. Indeed, members of the CHP protein family affect NHE1 activity and trafficking [26] and ezrin binding to NHE1 promotes remodeling of the actin cytoskeleton [60, 61] suggesting that a coordinate CHP1 and ezrin action to facilitate localized signal relay [62] might not be restricted to NHE3.

## Supporting Information

S1 File Proposed model of coordinate CHP1 and ezrin regulatory action on NHE3 activity. Two pathways of ezrin activation were tested (pathway 1 = binding of PIP2 to ezrin and pathway 2 = phosphorylation of ezrin; pathway 1 helps allow pathway 2) by expression of ezrin variants: Ezrin binding to PIP2 and its phosphorylation are both important to control NHE3 activity under baseline conditions (Figure A). CHP1 controls NHE3 activity via ezrin activation (Figure B). Our findings seem to exclude the possibility that CHP1 acts on ezrin activation via pathway 1, while possible mechanisms of CHP1 action on pathway 2 remain to be defined. Further studies are necessary to determine the role of CHP1 on pathway 2.
(TIF)
