# Supplementary figures and images for: The Na+/H+ Exchanger-3 (NHE3) Activity Requires Ezrin Binding to Phosphoinositide and Its Phosphorylation
Source: PLoS One. 2015 Jun 4;10(6):e0129306. doi: 10.1371/journal.pone.0129306 (PMC4455992; doi:10.1371/journal.pone.0129306)

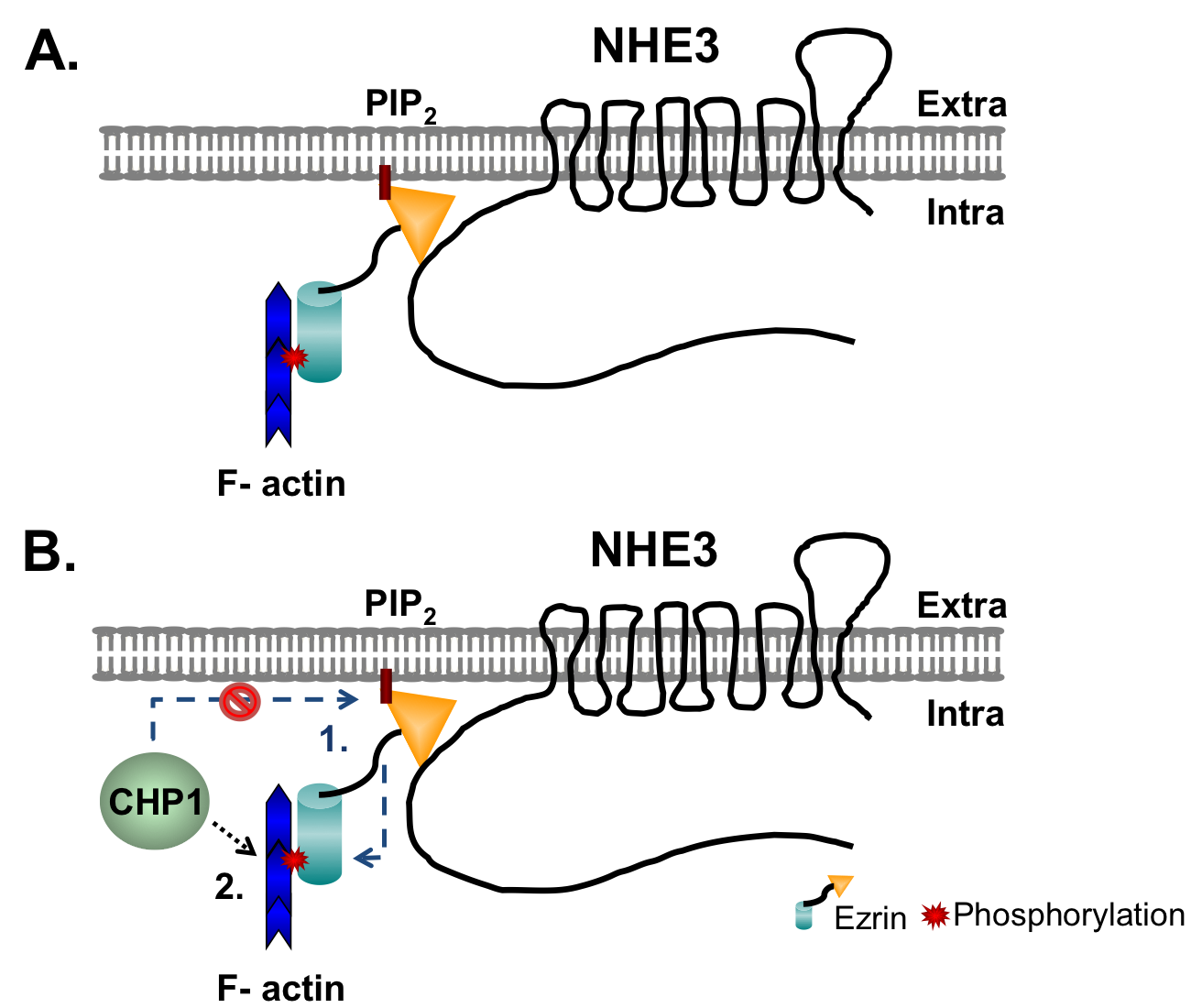

Supplement: S1 File — Two pathways of ezrin activation were tested (pathway 1 = binding of PIP2 to ezrin and pathway 2 = phosphorylation of ezrin; pathway 1 helps allow pathway 2) by expression of ezrin variants: Ezrin binding to PIP2 and its phosphorylation are both important to control NHE3 activity under baseline conditions (Figure A). CHP1 controls NHE3 activity via ezrin activation (Figure B). Our findings seem to exclude the possibility that CHP1 acts on ezrin activation via pathway 1, while possible mechanisms of CHP1 action on pathway 2 remain to be defined. Further studies are necessary to determine the role of CHP1 on pathway 2. (TIF) [file pone.0129306.s001.tif]
